# Immune system responses and fitness costs associated with consumption of bacteria in larvae of *Trichoplusia ni*

**DOI:** 10.1186/1741-7007-5-56

**Published:** 2007-12-21

**Authors:** Dalial Freitak, Christopher W Wheat, David G Heckel, Heiko Vogel

**Affiliations:** 1 Max Planck Institute for Chemical Ecology, Department of Entomology, Hans-Knoell – Strasse 8, 07745 Jena, Germany; 2 University of Helsinki, Department of Biological and Environmental Sciences, Helsinki FI-00014, Finland

## Abstract

**Background:**

Insects helped pioneer, and persist as model organisms for, the study of specific aspects of immunity. Although they lack an adaptive immune system, insects possess an innate immune system that recognizes and destroys intruding microorganisms. Its operation under natural conditions has not been well studied, as most studies have introduced microbes to laboratory-reared insects via artificial mechanical wounding. One of the most common routes of natural exposure and infection, however, is via food; thus, the role of dietary microbial communities in herbivorous insect immune system evolution invites study. Here, we examine the immune system response and consequences of exposing a lepidopteran agricultural pest to non-infectious microorganisms via simple oral consumption.

**Results:**

Immune system response was compared between *Trichoplusia ni *larvae reared on diets with or without non-pathogenic bacteria (*Escherichia coli *and *Micrococcus luteus*). Two major immune response-related enzymatic activities responded to diets differently – phenoloxidase activity was inhibited in the bacteria-fed larvae, whereas general antibacterial activity was enhanced. Eight proteins were highly expressed in the hemolymph of the bacteria fed larvae, among them immune response related proteins arylphorin, apolipophorin III and gloverin. Expression response among 25 putative immune response-related genes were assayed via RT-qPCR. Seven showed more than fivefold up regulation in the presence of bacterial diet, with 22 in total being differentially expressed, among them apolipophorin III, cecropin, gallerimycin, gloverin, lysozyme, and phenoloxidase inhibiting enzyme. Finally, potential life-history trade-offs were studied, with pupation time and pupal mass being negatively affected in bacteria fed larvae.

**Conclusion:**

The presence of bacteria in food, even if non-pathogenic, can trigger an immune response cascade with life history tradeoffs. *Trichoplusia ni *larvae are able to detect and respond to environmental microbes encountered in the diet, possibly even using midgut epithelial tissue as a sensing organ. Potential benefits of this immune system priming may outweigh the observed tradeoffs, as priming based on environmentally sensed bacterial may decrease risk of serious infection. These results show that food plant microbial communities represent a dynamic and unstudied part of the coevolutionary interactions between plants and their insect herbivores.

## Background

Herbivorous insects are one of the most diverse and successful groups of animals on earth, having been able to invade and exploit nearly every available ecological niche [1]. Having relatively short generation times and large numbers of progeny per adult allows insects to adapt quickly to various biotic and abiotic stressors in the environment, including pathogens. Invertebrate immunity studies have revealed valuable information on the induction and propagation of the immune response, focusing on the signaling cascades activated after pathogen recognition [2-6]. Immune responses are costly and result in trade-offs with other life-history traits, such as reproduction and development [7]. In most studies, lab reared insects have been infected with bacterial strains via artificial mechanical wounding (i.e. injection), neglecting the main routes of natural exposure to bacteria, most notably via plant consumption [8]. Thus, the role of plant microbial communities in herbivorous insect host use and performance is largely unknown.

Herbivorous lepidopteran larvae consume large quantities of plant material over the course of their development from neonate to late instar larvae, increasing as much as up to 20% of their total body weight per day [9,10]. Studies of host shifts onto novel host plants have traditionally focused on considerations of the new abiotic factors (eg. thermal, temporal) [11] and biotic conditions (e.g. competition, secondary plant metabolites) [1,12] to which the herbivore must adapt. However, new host plants could also harbor different, possibly pathogenic microorganisms [8,13]. Both the surface and the interior of the plant leaf are known to contain diverse and dense bacterial communities, which are distributed both as single cells and extensive biofilms [14]. Microbial communities are known to vary between the conspecific plants as well as between different leaves and parts of the same plant [8]. Therefore, larvae are naturally exposed to microbes via consumption and this diversity adds to the list of novel niche conditions to which herbivorous insects' immune system must adapt.

One of the major foci of the evolutionary ecology of immunity is the identification and understanding of the selective forces shaping and maintaining immune defenses, focusing on both the factors that induce an immune response and the consequences of that response [15]. The immune defense system of insects consists of behavioral barriers, passive defensive barriers (cuticles), and cascades of active responses that follow after cuticular injury and exposure of the hemocoel to pathogens [16,17]. Lacking an adaptive immune system, insects rely on an innate immune system, which controls most infections through inflammatory responses after pathogen recognition. This provides a potent first line immune defense resembling the innate immune system of mammals [18,19].

Antimicrobial peptides were first discovered in Lepidoptera [19], with the recognition, signaling, and antimicrobial peptide production of the innate immune system being subsequently determined in detail in *Drosophila melanogaster *and *Bombyx mori *[20,21,2]. Although antigen-specific antibodies are not produced by insects, an immune response to a later immune challenge can be enhanced by previous exposure [22-25]. In this case, the initial microbial encounter serves as immunological priming with specific hemolymph synthesized proteins remaining in circulation for weeks [26]. Epithelial tissues do appear able to recognize pathogens and express antibacterial protein encoding genes [8,27-31]. Thus, midguts would be expected to have the potential to sense microbial presence, which would allow a timely and relevant immune system priming. However activating the immune system can be costly, having consequences on other life-history traits [7,17].

Immune response costs of resistance, avoidance and tolerance towards pathogens can differ [7,32]. Ideally, depending on the probability and nature of microbes encountered in the environment, different host resistance mechanisms should be employed [32,33]. Thus, for efficient allocation of resources, an organism needs to be able to differentiate among pathogenic and nonpathogenic microorganisms and to react accordingly, i.e. to prime the immune system only when necessary. In the case of pathogens using the digestive tract as a gateway to infect the host or posing a threat in the local environment, the midgut could act as a sensing organ for priming the immune system [8,34]. A well-studied example for a specific host-parasite interaction is the work performed on the immune system and immune gene repertoires in the mosquito *Anopheles gambiae*. The relationship between insect and parasite has been finely tuned, enabling the parasite to partially evade the insect immune system, develop in the insect gut epithelium and then travel to the insect salivary glands [35-37]. Several mosquito genes have been identified that control the immune response of *Anopheles*, directly affecting development of the malaria parasite within the insect gut [38]. However, except for these very specific cases of host-parasite interactions, little is known about what kind of effect digested microbes, even naturally occurring essentially non-pathogenic microbes, have on insect immunological ecology.

Here, we examine the consequences of exposing insects to non-infectious microorganisms via simple oral consumption. The ability of an herbivorous insect, the cabbage semilooper *Trichoplusia ni *(Lepidoptera), to both detect and respond to non-pathogenic, non-infectious bacterial communities through normal consumption, as well as the potential fitness consequences of such a response are studied. Comparisons were made between sterile artificial diet vs plant feeding and artificial diet supplemented with both gram positive and gram negative bacteria. Immune system response was assayed with a detailed analysis of artificial diet treatments at three different levels: enzyme activity, mRNA expression, and protein levels. Fitness consequences were observed for two key indicators of fitness parameters – larval maturation rate and pupal weight – to be correlated with presence or absence of bacteria in the food, indicating a significant cost to the immune system induction. We believe this to be the first study of the ability of ingested non-pathogenic, non-infectious bacteria as inducers of invertebrate immune system responses in lepidopteran larvae. Ingested non-pathogenic bacteria can upregulate lepidopteran immune genes, with consequences, and our results suggest this effect may be important in host disease resistance.

## Results

### Enzyme activities in the hemolymph

Hemolymph samples were collected from 9-day-old *T. ni *larvae grown on bacterial and bacteria-free diet. We measured enzyme activities for two commonly used immune status indicators in insect immunology studies – general antibacterial and phenoloxidase activity. For estimating the differences in general antibacterial activity we used standard lytic zone assays. Significant differences were found in the general antibacterial activity and phenoloxidase activity of the hemolymph depending on the type of diet in *T. ni *larvae.

**Figure 1:**
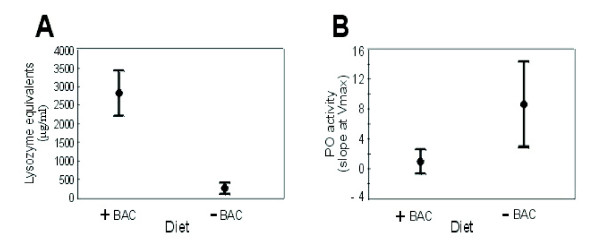
**Enzyme activities in the hemolymph of last instar *T. ni *larva, fed on bacteria-free (-BAC) and bacteria-supplemented diet (+BAC)**. (A) General antibacterial activity measured as the diameter of the lytic zone on agar plates and transformed into lysozyme equivalents (μg/ml). (B) Phenoloxidase activity (slope at Vmax) measured from hemolymph samples. Results represent mean values ± SE.

Adding bacteria to the artificial diet lead to higher lysozyme activity in comparison to larvae grown on the diet without bacteria (Kruskall-Wallis ANOVA; H_1,58 _= 7.77; p = 0.003) (Figure 1A). Immune induction can generally lead to an increase of the titer of antibacterial proteins and peptides in the hemolymph. These are usually lytic enzymes (e.g. lysozyme) causing bacterial cell wall degradation, and small pore forming peptides leading to the lysis and leakage of the bacterial membranes. The antibacterial 'cocktail' measured by lytic zone assays may consist not only of different lysozymes but also of unknown lytic and antibacterial proteins.

In contrast, bacterial diet had a negative effect on hemolymph phenoloxidase, as animals fed on bacteria-free diet had a significantly higher steady state activity as compared to larvae fed with bacteria (Kruskall-Wallis ANOVA; H_1,130 _= 31.39; p = 0.000) (Figure 1B). Arthropod melanisation is controlled by a cascade of serine proteases that ultimately activates prophenoloxidase (PPO) to the enzyme phenoloxidase (PO), which, in turn, catalyzes the synthesis of melanin and is widely used as an estimate of immunocompetence. The PO level is believed to be in good correlation with insects' immunocompetence, especially against invading fungi or insect parasitoids [39-42].

### Identification of differentially expressed proteins in the hemolymph

**Figure 2:**
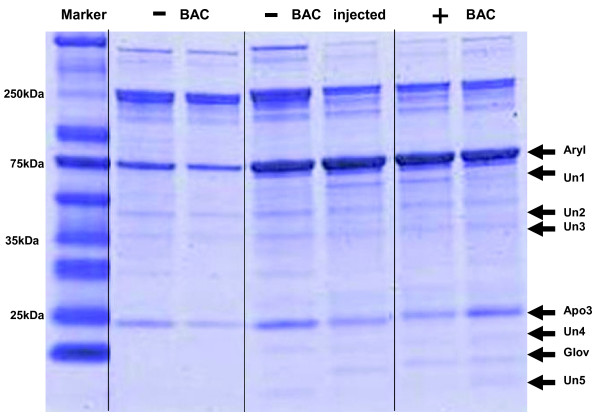
**SDS gel electrophoresis of *T. ni *hemolymph proteins stained with Coomassie blue**. Treatments are bacteria-free diet (-BAC), injection of bacteria into hemocoel of larvae fed bacteria-free diet (-BAC injected), or bacteria-supplemented diet (+BAC). Duplicate lanes of each of the three treatments are shown. Identified differentially expressed proteins are arylphorin (Aryl), apolipophorin III (Apo3) and gloverin (Glov). Five additional unknown proteins (Un1, Un2, Un3, Un4, Un5) were observed as differentially expressed.

On one-dimensional protein gels (1D SDS-PAGE), we observed increased expression of eight proteins in the hemolymph of bacterial diet fed larvae and plant-fed (data not shown) as compared to larvae from bacteria-free diets (Figure 2). This pattern of increased expression was very similar to that produced by hemocoel injection of bacteria into larvae fed bacteria-free diets. For the identification of induced hemolymph proteins, tryptic digests were performed, peptide mass mapping using MALDI-TOF mass spectrometry was carried out, and de novo sequencing of peptides conducted by nano LC-MS/MS. To complete the searches, tandem mass spectra were interpreted *de novo *and the obtained sequences were used for MS-BLAST database searches. Three proteins out of eight were identified: arylphorin, apolipophorin III and gloverin. Most of the unidentified gel bands are very small proteins, and failure of identification is potentially related to extraction and digestion during sample handling and general limitations of MS in identifying very small proteins. Identified peptides and their relative position within the protein sequences are shown in Additional file 1.

All three identified proteins are thought to participate in immune responses. Arylphorin, for which we observed the greatest up-regulation of protein in the hemolymph, has been proposed to play a role in humoral immune defense in response to bacterial challenge [43-45]. Additional assays were performed to confirm the connection of higher arylphorin expression with larval immune challenge, ruling out the possible effect of nutritional differences between bacterial vs bacteria-free diets. Larvae grown on bacteria-free diet were injected with the mixture of the *E. coli *and *M. luteus *and a similar up-regulation of arylphorin expression was seen as in bacterial diet fed larvae (Figure 2). Injecting bacterial diet fed larvae with the bacterial mixture or saline did not cause any additional increase in arylphorin expression (data not shown). Second, larvae on bacterial diet also had a higher protein expression of apolipophorin III in the hemolymph. Apolipophorin belongs to the functionally important family of apolipoproteins that play critical roles in lipid transport and lipoprotein metabolism [46]. The third protein identified as being differentially expressed was gloverin. Gloverin is an inducible antibacterial insect protein first isolated from the silk moth *Hyalophora *[47]. It is a small, basic, heat stable protein containing a large numbers of glycine residues, but no cysteine residues as is found in many other antimicrobial peptides (e.g. defensin). Gloverin was also previously described to be expressed upon immune insult in *T. ni *and *Bombyx mori *[48,49].

### EST analysis and identification of immune-related genes in *T. ni*

For many lepidopteran species, including *T. ni*, only a very limited number of sequences are available in public databases. To identify immune-related and general housekeeping genes in larval tissues, a cDNA library was constructed from whole *T. ni *larvae of different instars and fed a combination of dietary inducers (i.e. plant secondary metabolites). DNA sequencing from the 5' ends of clones followed by clustering produced 1 675 distinct genes, 1 082 represented by single reads. For putative functional assignments, the assembled sequences were compared against protein and nucleotide NCBI databases, using the locally installed BLAST search tool.

BLAST searches and annotation using Gene Ontology terms showed that several ESTs were similar to known immune-related genes from other insects, including genes involved in pathogen recognition (pattern recognition proteins), direct antimicrobial defense (antimicrobial peptides) and genes related to physiological changes upon immune challenge. Among the ESTs were several known immunity-related genes (lysozyme, gloverin, pro-phenoloxidase) and several genes with similarities to immune-induced genes (HDD1, hemolin) with a total of 25 immune candidate genes. BLAST results and putative functions for the immune-related genes identified from *T. ni *are listed in Table 1.

**Table 1:** Immunity-related products discovered from *T. ni *EST projects

**TC/EST name**	**No. of ESTs present**	**Best BLAST hit/Closest homologue [Species]**	**Description/Putative Function**	**Accession no.**	**BLAST score (E-value)**
TNI-CON0233	23	Apolipophorin-3 precursor (Apolipophorin-III) [Manduca sexta]	Lipid transport; immune stimulating factor	P13276	1.00E-75
TNI-CON0998	1	Apolipophorin-3 precursor (Apolipophorin-III) [Spodoptera littoralis]	Lipid transport; immune stimulating factor	O77248	7.00E-15
TNI-CON0275	3	Large subunit arylphorin p76 [Heliothis virescens]	Storage protein; expressed after immune challenge	AAO20844	3.00E-77
TNI-CON0268	1	Chemosensory protein 11 [Bombyx mori]	GNBP-like domain; immune responsive	NP_001037068	1.00E-33
TNI-HCN384-03G20	4	Beta-1,3-glucan-binding protein 2 precursor (BGBP-2) [Manduca sexta]	Bacterial cell wall binding/recognition protein	Q8ISB6	5.00E-52
TNI-CON1186	1	Beta-1,3-glucan-binding protein precursor (BGBP) [Plodia interpunctella]	Bacterial cell wall binding/recognition protein	Q8MU95	2.00E-31
TNI-CON0313	1	Beta-1,3-glucan recognition protein [Plodia interpunctella]	Bacterial cell wall binding/recognition protein	AAM95970	4.00E-38
TNI-CON1099	2	Beta-1,3-glucan-binding protein precursor (BGBP) [Plodia interpunctella]	Bacterial cell wall binding/recognition protein	Q8MU95	1.00E-28
TNI-CON0703	3	KUN-5 [Ixodes pacificus]	Kalicludin-like; Kunitz family of serine protease inhibitors	AAT92116	6.00E-12
TNI-HCN384-03D11	2	Kunitz-like protease inhibitor precursor [Ancylostoma caninum]	Kalicludin-like; Kunitz family of serine protease inhibitors	AAN10061	2.00E-10
TNI-CON0522	1	Phenoloxidase inhibitor protein [Anopheles gambiae]	Inhibition of phenoloxidase cascade	AAX22219	7.00E-05
TNI-CON1448	1	Prophenol oxidase activating enzyme 1 [Spodoptera litura]	Activating enzyme of Pro-PO	AAW24480	7.00E-46
TNI-CON0527	3	Conotoxin scaffold VI/VII precursor [Conus arenatus]	Conotoxin-like protein; ion channel antagonist	AF215057	7.00E-05
TNI-CON0581	1	Gallerimycin [Spodoptera frugiperda]	Defensin-like antifungal peptide	AAQ18896	2.00E-20
TNI-CON1119	1	Putative hemolin [Hyphantria cunea]	Immunoglobulin domains; induced by microbial challenge	AAD09287	4.00E-51
TNI-HCN384-02J03	3	Putative hemolin [Hyphantria cunea]	Immunoglobulin domains; induced by microbial challenge	AAD09287	4.00E-85
TNI-CON0811	1	Attacin-A precursor [Trichoplusia ni]	Inducible antibacterial peptide	P50725	2.00E-22
TNI-CON0122	22	Cecropin D [Bombyx mori]	Antimicrobial peptide; Lysis of bacterial cell walls	BAA31507	9.00E-10
TNI-CON0128	9	Cecropin [Helicoverpa armigera]	Antimicrobial peptide; cecropin B	AAX51304	1.00E-16
TNI-CON0196	2	Immune-related Hdd1 [Hyphantria cunea]	Immune-related protein; induced by microbial challenge	AAD09279	4.00E-09
TNI-CON0498	2	Defensin precursor [Spodoptera frugiperda]	Antimicrobial peptide; spodoptericin-like	AAM96925	5.00E-21
TNI-CON0644	4	Cobatoxin short form A [Spodoptera frugiperda]	Scorpion toxin-like; Induced after bacterial challenge	AAQ18897	5.00E-07
TNI-CON1679	2	Gloverin precursor [Trichoplusia ni]	Antibacterial protein; binds to LPS	AF233590	8.00E-51
TNI-CON0507	3	Lysozyme [Spodoptera exigua]	Lysozyme a; destroys bacterial cell walls	AAP03061	3.00E-44
TNI-CON1157	1	Lysozyme precursor [Trichoplusia ni]	Lysozyme b; destroys bacterial cell walls	P50718	1.00E-36

### RT-qPCR of immune-related genes in *T. ni *midgut tissue

Transcript profiles from *T. ni *larvae fed on bacterial and bacteria-free diet were compared using quantitative real-time PCR. Experiments were conducted each with three biological replicates and randomized between the treatments. A total of 28 gene-specific primer pairs (see Additional file 2 for primer information) were designed on the basis of sequences obtained for selected *T. ni *genes known to be involved in immune response in other insect species or were based on proteins identified as immune-responsive in our protein expression experiments. In addition, elongation initiation factor-4α (EIF4α), elongation factor-1α (EF1α) and mitochondrial ribosomal protein S18 (RPS18) were selected to serve as potential housekeeping genes. All three were tested as invariant endogenous controls in the assay to correct for sample to sample variation in RT-qPCR efficiency and errors in sample quantitation and sample concentration. EIF4α performed best as an endogenous control ('normalizer') and was used for the remaining assays.

**Figure 3:**
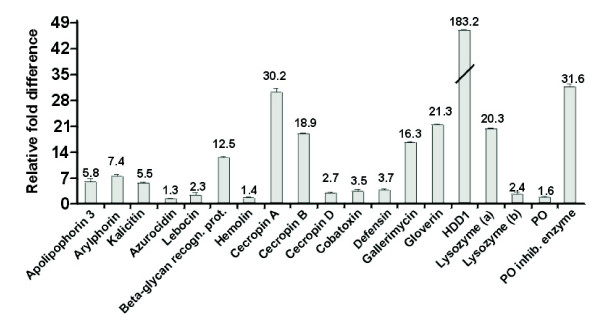
**RT-qPCR results of differential gene expression between *T. *ni larvae grown on bacterial diet in comparison to larvae grown on bacteria-free diet**. Relative fold changes for each gene were set to 1 for the control treatment. Results represent mean values of three independent biological replicates ± SD (experimental error).

Relative fold changes for each gene were set to 1 for the control treatment (larvae grown on bacteria-free diet). Seventeen genes were up-regulated by bacterial feeding, including apolipophorin III, arylphorin, cecropin B, cecropin D, cobatoxin, defensin, gallerimycin, gloverin, HDD1, kalcitin, lysozyme(a+b), a β-1,3-glucan recognition protein, and phenoloxidase inhibiting enzyme. Apolipophorin III, arylphorin, a β-glycan recognition protein, cecropin B, HDD1 immunity related protein and lysozyme(a) were more than fivefold up-regulated in the midguts of larvae grown on bacterial diet (Figure 3). Notably, lysozyme(a), which is highly upregulated at the transcript level, was also identified by MS as being upregulated at the protein level. No statistically significant changes in transcript abundance by bacterial feeding could be detected for azurocidin, attacin and a Gram negative-binding protein.

### Life-history traits

**Figure 4:**
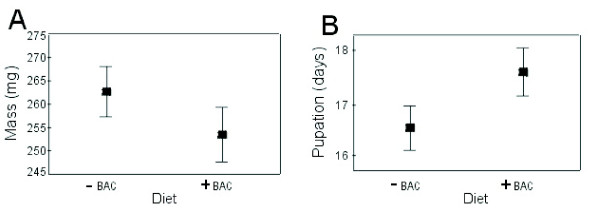
**The effect of bacterial diet (+BAC) and bacteria-free diet (-BAC) on the pupation time and pupal masses in *T. ni***. Graphs shows least square means of the model, representing mean ± SD. (A) Pupal masses are decreased when larvae are fed bacterial diet in comparison to larvae grown on bacteria-free diet. (B) Bacterial diet leads to delayed pupation times in *T. ni *larvae.

To examine whether the observed changes in protein expression and enzyme activity in the hemolymph, as well as differential gene expression in the gut, have any life history consequences, we examined the larval developmental time and pupal mass of animals grown on the different diets. Animals grown on bacterial diet had smaller pupal masses and delayed development. The complete life cycle of *T. ni *from egg to adult death under the conditions used for our experiments is approximately 4–6 weeks, with the larval stage lasting, on average, 2 weeks. Bacteria fed larvae reached the pupation state 1–1.5 days later than larvae grown on bacteria-free diet (ANOVA; F_1,204 _= 11.16, p = 0.001) (Figure 4B). The diet did not influence the developmental time of the two sexes differently (ANOVA; F_1,143 _= 0.03, p = 0.858). Animals grown on bacterial diet also had smaller pupal masses in comparison to animals grown on bacteria-free diet (ANOVA; F_1,143 _= 9.77, p = 0.002) (Figure 4A). Again, diets had no differential effect on sex, as the trend for mass loss was the same for males and females on both diets (ANOVA; F_1,143 _= 0.08, p = 0.778). The bacterial-fed *T. ni *larvae, though having a longer developmental time, fail to reach the same pupal mass as conspecifics grown on the bacteria free diet. We could not observe any differences in mortality, failure to pupate or development into adults between the treatment groups.

## Discussion

Insects possess a range of defense mechanisms to effectively combat invasion by microbial pathogens. Here we document for the first time that ingested non-pathogenic bacteria can induce an immune response in invertebrates with fitness related costs. These effects can be seen for both direct and indirect immune responses. A direct response was observed as bacterial diet altered two important immunity related functions of the hemolymph.

Phenoloxidase (PO) activity is widely used as an indicator of insects' immunocompetence [50-52,40]. In *T. ni*, bacterial diet has an inhibiting effect on PO activity in the hemolymph. However, at the same time the overall antibacterial lytic activity of the hemolymph was significantly increased. The lytic activity usually consists of a cocktail of small lytic enzymes causing bacterial cell wall degradation, leading to the lysis and leakage of the bacterial membranes. Lysozymes and other lytic proteins can be active against both Gram-positive and Gram-negative bacteria, potentially activating the Toll-related signaling pathway by releasing bacterial cell wall material [2,16]. Opposite responses of general lytic and PO activity could represent a trade-off between different types of immune responses, correlated with the nature of the immune induction. Lysozyme activity is usually more related to bacterial infection and PO activity to fungal and multicellular hemolymph-invading organisms. A trade-off between PO and lysozyme activity has been reported in larvae from the related Noctuid moth *Spodoptera littoralis *[53]. It is unclear whether these apparent tradeoffs, observed both here and the previous study, arise from similar induction pathways, limited resources, or a mechanistic trade-offs due to the potentially severe harmful side effects of high PO activity on the tissues in the form of oxidative and lytic stress [54]. Further studies at the individual level, more directly focused on this apparently general phenomena, are now warranted.

Initial experiments performed with larvae fed on *Brassica *plants grown in the greenhouse provided intermediate results for both the lytic zone assays and the phenoloxidase activities (data not shown). However, variations in plant secondary metabolites potentially interfere or overlap with results obtained from bacterial community variations. This is also reflected in the high overall variability in the responses of *T. ni *larvae placed on individual leaves or plants. Using plants as a food source does not facilitate clear differentiation between effects caused by leaf surface bacteria and plant secondary metabolite variations. In order to exclude effects due to secondary plant chemistry, we focused our research on artificial diet manipulation.

The observed increase in transcript abundance of a ProPO inhibiting enzyme in the midgut tissue of *T. ni *larvae fed on bacterial diet could potentially contribute to the lower overall PO activities in the hemolymph. Lower levels of PO activity in the hemolymph could also be related to up regulation of apolipophorin III expression in the plasma, as has been shown in the wax moth (*Galleria mellonella*) [55].

We observed an increase in arylphorin, apolipophorin III, and gloverin protein levels in hemolymph, as well as an increase in transcript abundance in the midgut, in larvae fed bacterial-supplemented diet. Arylphorin is usually highly abundant during the last larval instars, but its synthesis ceases during the molt, during starvation, and at the wandering stage. Arylphorin is one of the major storage proteins, has been proposed to play a role in humoral immune defense as a response to bacterial challenge but is also known to be either more abundant after parasitization by wasps [39] or dramatically reduced relative to the levels of arylphorin detected in nonparasitized larvae [44,45,56,57]; however its exact function in immune response has not been clearly established.

Apolipophorin III (ApoIII) belongs to the functionally important class of lipoproteins, which are responsible for lipid transport and lipoprotein metabolism in various animal classes [46]. ApoIII seems to be an insect-specific protein as it has not been described in vertebrates [4]. The immune response related properties of ApoIII were first described in greater wax moth (*Galleria mellonella*) [58]. Although ApoIII has mainly been described to be a storage protein, it was also shown to have general immune stimulating activity and to bind bacterial lipoteichoic acid. Arylphorin is generally able to bind to molecules characteristic to microorganisms, classifying it as a pattern recognition protein, involved in sensing the presence of bacteria, or in a more protective role by neutralizing pathogen cell wall components [59,60,55,46]. Besides having a general immune stimulating activity, injection of ApoIII as well as *E. coli *in *Hyphantria *dramatically induced the expression of antimicrobial peptides, and as previously mentioned, repressed PO activity [53,59], which is consistent with our findings.

We have also detected high levels of the immune-related effector protein gloverin in the hemolymph of bacteria fed larvae. Gloverin is an antibacterial protein, and was shown to be synthesized after bacterial immune challenge, being active against Gram-negative bacteria and yeast in *Helicoverpa armigera *[61], inhibiting the growth of *E. coli *at concentrations far below the concentration found in the hemolymph of infected pupae [47]. It is also expressed in *T. ni *hemocytes following bacterial induction [48]. The prime effect of gloverin seems to be binding to lipopolysaccharides on the bacterial outer membrane, inhibiting synthesis of essential outer membrane proteins, leading to increased permeability [47]. Notably, a similar spectrum of the induction of immune-responsive proteins was identified in *Galleria mellonella *by using comparative proteomic analyses of hemolymph proteins and RT-qPCR analysis from larvae that were challenged with either injecting microbial metalloproteases or LPS [62].

The increase in transcript abundance of several immune response related genes in midgut tissues suggests that midgut cells themselves are able to recognize and respond to the presence of bacteria in the gut lumen. The innate immune response of epithelial cells has been studied rarely [27,28]. *T. ni *larvae feeding on bacteria supplemented diet show higher expression of an azurocidin-like protein in the gut [29]. Experiments performed with *Drosophila melanogaster *show that the expression of a drosomycin-GFP reporter gene in epithelial tissues responds to infection [28]. Furthermore, some bacterial species are able to trigger a strong systemic immune response in Drosophila after oral infection, possibly mediated through a peptidoglycan receptor protein (PGRP-LB), which was suggested to being activated only in the case of severe infection and bacterial proliferation [34]. We know of only one study showing that non-pathogenic bacteria can induce immunity-related genes after oral feeding, with honey bees showing expression differences in a single gene coding for an antimicrobial protein (abaecin) after feeding on a bacterial mix [63]. However, the consequence of an epithelial immune response for the systemic immune response, immunocompetence and other life-history traits is largely uninvestigated.

Two important life-history traits were affected by the consumption of bacterial diet. The increase in developmental time and decrease in pupal mass observed in the bacterial-fed treatments are both likely to have negative effects on overall fitness. We have used a nutrient-rich diet, providing optimal conditions for both larval growth and development. Costs in the wild are likely to be greater due to the likely substandard abiotic and biotic conditions. Moreover, the opportunity for additional growth during the increased larval period of approx 1.5 day was not sufficient to compensate for the reduced growth rate, as bacterial-fed individuals failed to reach the same pupal mass as conspecifics reared on nonsupplemented diet. For both the pupal mass and developmental time, the diet had no differential effect on sex, as the trend for mass loss was the same for males and females on both diets. Furthermore, we could not observe any differences in mortality, failure to pupate or development into adults between the treatment groups. This would support the idea that, although costly due to immune priming, bacterial-supplemented diet did not have any direct deleterious effects reflected in the survival of the insects.

Negative fitness related effects have been reported also for the larvae of the gypsy moth (*Lymantria dispar*) infected with the entomopathogenic microsporidium *Vairimorpha *sp (Microsporidia: Burenellidae). These larvae have prolonged development due to decreased food utilization, also resulting in a decreased body mass [64]. In mosquito (*Aedes aegypti*), malarial infection reduces the fecundity, increases mass loss, and lowers metabolic rate during food digestion [65]. In our experiments and as an important contrast, nonpathogenic bacteria without infection (bacteria not being present in the hemolymph) resulted in comparable effects, which we interpret as a cost of 'priming' the innate immune system.

Such an anticipatory up-regulation of immune defenses can have benefits as well, as illustrated in a recent study. In *Manduca sexta *larvae, prior hemolymph injection of non-pathogenic bacteria elicited up-regulation of several genes, which provided some protection against subsequent infection with pathogenic *Photorabdus*. These protective effects were weakened by experimental manipulation of transcript levels by RNA interference [66]. These findings support the adaptive significance of a 'priming' of the immune system, leading to a higher level of immune responses that would enable the insects to better cope with real pathogens they may encounter. Moreover, pathogenicity may be context-dependent and 'priming' may actually be directly defensive against the entire bacterial community experienced by an insect. Broderick *et al.*[67] have shown that the insecticidal activity of *Bacillus thuringiensis *is dependent on interactions with other microorganisms of the larval midgut. Eliminating most of the midgut bacteria drastically reduced larval mortality even in the presence of the insecticidal crystal protein of Bt, suggesting a complex interaction of non-pathogenic and pathogenic bacteria.

Our results suggest that the midgut may play a more active role in sensing foreign organisms and mounting protective responses than previously suspected. Moreover, immune-related properties of the hemolymph may be affected even if the foreign organisms never enter the hemocoel. The nature of the signal and the mechanisms for modulating a gut signal into a hemolymph and fat body response remains unclear and is likely to be a rewarding avenue of research. Dissected *T. ni *larvae exposed to non-pathogenic bacteria in their diet have no detectable lesions in the peritrophic matrix or epithelium. Preliminary feeding tests performed with fluorescently labeled bacteria also indicate that no marker can be detected in the hemolymph (data not shown), although we cannot rule out the possibility of bacterial fragments crossing the gut wall. Expression of both pattern recognition and antimicrobial proteins by midgut cells points to the potential of recognizing and fighting bacteria directly in the gut tissue and gut lumen. Thus, the midgut deserves attention not only as an organ of digestion and resource assimilation, but also of defense. Further studies will lead to the molecular characterization of receptor molecules and signal transduction pathways involved in guarding this vulnerable portal.

## Conclusion

This work has addressed the consequences of exposing insects to non-infectious microorganisms via simple oral consumption. Here, we show that larvae can sense microbes through consumption, as hemolymph specific defense mechanisms can be induced without actual exposure to and infection with microorganisms. Nonpathogenic bacteria in larval food induce specific changes in the larval proteome, transcriptome and enzyme activity levels. Although such physiological changes negatively affect fitness related traits, such as body mass and developmental time, the potential benefits of immune system priming may outweigh the observed tradeoffs, as priming based on environmentally sensed bacteria may decrease the risk of serious infections. These results strongly suggest that host plant microbial communities may represent a dynamic and unstudied part of the evolutionary interactions between plants and their insect herbivores.

## Methods

### Animals

Cabbage semilooper (*Trichoplusia ni*) eggs were obtained from Entopath Inc. (Easton, PA, USA). Larvae of *Trichoplusia ni *were grown on artificial diet (casein 31.5 g, sucrose 33.76 g, wheat germ 43.76 g, Wess salt 9 g, potassium sorbate 1 g, cellulose 6.26 g, methyl paraben 1.36 g, lepidopteran vitamin mix 9 g, aureomycin 1 g, ascorbic acid 3.5 g, propyl gallate 0.2 g, 40% formaldehyde 1.5 ml, linseed oil 6.5 ml, 45% potassium hydroxide 2.5 ml, 24 g agar and 750 ml water) at room temperature (23°C) and a 16/8 h light/dark cycle, and 55% relative humidity. For initial tests, larvae were also reared on cabbage plants (*Brassica oleracea oleracea*, var. Rosella). Seeds from Brassica plants were sown on a mini-tray: vermiculite (3:1) soil mix (Einheitserdenwerk, Froendenberg, Germany) and cold stratified for 7 days at 4°C. Afterwards, plants were moved to the greenhouse and grown at 23°C with fluorescent light banks with wide spectrum lights.

*T. ni *eggs were either placed in plastic cups with artificial diet or on 3-week-old plant leaves and allowed to hatch. To estimate the impact of bacteria in the diet, three feeding groups were formed: larvae were fed on artificial diet with or without bacteria (later referred to as bacterial and bacteria-free diet) and for initial experiments on *Brassica *plants. Bacterial diet was soaked with overnight cultures (OD600 = 4) (2.5 ml/40 cm^2^) of *Escherichia coli *and *Micrococcus luteus *(approximately 80 μg per 125 g of diet). Diets were changed every 3 days to keep the bacterial concentration in the diet at approximately the same level. In the case of growth rate experiments, larvae were kept in individual cups (~30 ml) with a piece of artificial diet and pupation was estimated on a daily basis. Pupal weight was measured using an electric balance to the nearest mg on the third day after pupation. For injection control experiments, 3 μl of saline (control) and *E. coli *and *M. luteus *(induction) pelleted cells in saline were injected into 9-day-old last instar larvae of both dietary groups using a FemtoJet microinjector (Eppendorf, Wesseling-Berzdorf, Germany).

Enzyme activities and protein expression in the hemolymph were measured from early stage last instar larvae (ninth day after hatching from egg). Same age last instar larvae were also dissected and their midguts removed and stored in RNA stabilizing buffer (Qiagen) for gene expression analyses.

### Lytic zone assay

For estimation of the total lytic activity of the hemolymph, a lytic zone assay was performed. A total of 12 × 12 cm Petri dishes were filled with 35 ml of autoclaved Sörensen buffer with 21 mg *Micrococcus luteus *lyophilisized cells (Sigma) and 2.1 mg streptomycin sulfate (Calbiochem, Bad Soden, Germany) with a final concentration of 1.5% agar. Wells within plates (2 mm diameter) were made by puncturing the agar with a plastic pipette and removing the agar plug by suction. Hemolymph samples (3 μl) were pipetted directly into the wells and the plates were incubated for 24 h at 37°C. Dilution series of chicken egg white lysozyme (Sigma, Seelze, Germany) (2 mg/ml, 1 mg/ml, 0.750 mg/ml, 0.500 mg/ml, 0.250 mg/ml, 0.125 mg/ml, 0.62 mg/ml, and 0.31 mg/ml) was added to each plate as a control and a calibration curve was created based on these standards. Lytic activity was determined as the radius of the clear zone around a sample well.

### Phenoloxidase activity assay

Hemolymph phenoloxidase activity was estimated using 10 μl of hemolymph sample diluted in 1 ml of ice-cold sodium cacodylate buffer (0.01 M Na-cacodylate and 0.005 M CaCl_2_) and directly frozen in liquid N_2_. PO activity was assayed by thawing frozen hemolymph samples at 37°C for 4 min and then centrifuged at 4°C and 2 800 *g *for 15 min. The supernatant was removed and used for measurements where 100 μl of supernatant was added to 200 μl of 3 mM L-Dopa (Sigma). Kinetic activity of the enzyme was measured at 30°C, 490 nm for 45 min, taking absorbance measurements once per min. As the absorbance curve was linear from 5–45 min after adding the substrate (D. Freitak, personal observation), in later analyses the slope of the curve from 15–26 min of the reaction was used. Measurements were made on Multiskan Spectrum multiplate reader (Thermo-Electron, Dreieich, Germany) and data was acquired with SkanIt Software for Multiskan Spectrum version 2.1 (Thermo-Electron).

### Protein gel electrophoresis and protein identification by MALDI-MS and NANOLC-MS/MS

To estimate protein expression in the hemolymph, sodium dodecyl sulphate polyacrylamide gradient gel electrophoresis (SDS-PAGE) was performed in a XT-MES buffer system. A total of 2 μl of hemolymph sample was diluted into 50 μl of ice-cold 4% SDS containing TrisHCl buffer with EDTA-free protease inhibitor cocktail (Pierce, Bonn, Germany), directly frozen in liquid N_2 _and stored at -20°C until use. For measurements, samples were allowed to melt on ice and centrifuged at 9 200 *g *for 10 min. Supernatant was transferred to new tubes, loading buffer was added to the supernatant, heat denatured and loaded on a 4–12% Bis-Tris Criterion XT Precast Gel (BioRad, München, Germany). Gels were run at 80 V for ~3.5 h or until the dye front reached the gel end. On the gels, two different protein markers were used. Rainbow marker (Amersham, Freiburg, Germany) served as a running control marker and the Precision Plus Protein Unstained Standard (BioRad) for precise protein molecular weight estimation. After the run was complete, gels were washed three times, followed by staining with Coomassie blue (Imperial Blue, Pierce) for 2–3 h, then destained overnight. For protein identification, spots were manually cut out from SDS-gels, transferred to 96-well microtiterplates (MTP) and processed on an automatic Ettan TA Digester (GE Healthcare, Freiburg, Germany). The gel plugs were rinsed with 50 mM ammonium bicarbonate/50% acetonitrile three times for 20 min to remove the coomassie stain. The gel plugs were then air-dried and digested with trypsin overnight at 37°C. The resulting peptides were extracted from the gel plugs, collected in a MTP and vacuum-dried. Samples were submitted for MALDI-TOF mass spectrometry and denovo sequencing by Q-TOF to our in-house MassSpec service group (MPI Jena, Germany).

For further processing, a MALDImicro MX mass spectrometer (Waters, Eschborn, Germany) was used for monitoring of the protein digestion. The tryptic peptides were reconstituted, mixed with α-cyano-4-hydroxy cinnamic acid, and an aliquot of the mixture was spotted on a metal 96-spot MALDI target plate. MassLynx v4.0 software served for data acquisition (Waters). Bovine serum albumin tryptic digest was used to calibrate the mass spectrometer (MPrep, Waters). The MALDI-TOF peptide signal intensities were used to estimate the volume of the sample for the nanoLC-MS/MS *de novo *sequence analysis.

Liquid chromatography-tandem mass spectrometry was performed to acquire fragmentation data from selected peptides. Aliquots of tryptic peptides were injected on a CapLC XE 2D nanoLC system (Waters). After concentration and desalting, eluted peptides were transferred to the NanoElectroSpray source of a Q-TOF Ultima tandem mass spectrometer (Waters). MS/MS spectra were collected by MassLynx v4.0 software (Waters). ProteinLynx Global Server Browser v.2.2 software (PLGS 2.2, Waters) was used for baseline subtraction and smoothing, deisotoping, *de novo *peptide sequence identification, and database searches. Obtained chromatograms were analyzed using the NCBI Insecta database [68] for MALDI – TOF samples and Swissprot database for Q-TOF samples. Amino acid sequences of peptides that did not provide conclusive results from the database searches were searched using an MS-BLAST server installed in-house or via the ButterflyBase web page [69]. Details of both the sample processing and instrument settings and handling have been described elsewhere [70].

### Preparation of *T. ni *cDNA libraries

For RNA isolation from larval tissue, in total 15 male and 15 female third, fourth and fifth instar larvae each were dissected in 100 mM Tris-HCl, pH 7.5. RNA and poly(A)+ mRNA was isolated with standard methods. Double-stranded, full-length enriched cDNA from dissected and whole larvae were generated by primer extension with the SMART cDNA library construction kit (Clontech, Heidelberg, Germany) according to the manufacturer's protocol but with several modifications. A total of 2 μg of poly(A)+ mRNA was used for each cDNA library generated. cDNA size fractionation was performed with SizeSep 400 spun columns (GE Healthcare) that resulted in a cutoff at ~300 bp. The full-length-enriched cDNAs were cut with SfiI and ligated to the SfiI-digested pDNR-Lib plasmid vector (Clontech) instead of the λ TriplEx2 vector provided with the kit. Ligations were transformed into *E. coli *ELECTROMAX DH5α-E electro-competent cells (Invitrogen).

### Generation of a *T. ni *EST sequence database

Plasmid isolation from bacterial colonies grown in 96 deep-well plates was performed using the 96 robot plasmid isolation kit (Eppendorf) on a Tecan Evo Freedom 150 robotic platform (Tecan, Crailsheim, Germany). Single-pass sequencing of the 5'-termini of a total of approximately 5 600 clones of the directionally cloned *T. ni *cDNA libraries was carried out on an ABI 3730 × l automatic DNA sequencer (PE Applied Biosystems, Weiterstadt, Germany). Vector clipping, quality trimming and sequence assembly was performed with the Lasergene software package (DNAStar, Madison, WI). Of the total of 5 300 ESTs, 770 were removed during the quality trimming steps. The average readable insert length after vector clipping and quality trimming was 545 bp. Blast searches were conducted on a local server using the National Center for Biotechnology Information (NCBI) blastall program. Sequences were aligned using ClustalW software [71]. *T. ni *sequences were submitted to Genbank under accession numbers EF605248, EU016384–EU016407.

### RNA isolation and quantitative real-time PCR

All larvae were 9 days old at the time of dissection. Dissected insect midguts were rinsed with PBS, ground using a motorized hand pestle and total RNA was isolated using the TRIzol Reagent (Invitrogen, Karlsruhe, Germany) according to the manufacturers' protocol. An additional DNAse (Turbo DNAse, Ambion, Darmstadt, germany) treatment was included prior to the second purification step to eliminate any contaminating DNA. A second purification step was performed with RNeasy MinElute columns (Qiagen, Hilden, Germany). RNA integrity was verified on an Agilent 2100 Bioanalyzer using RNA Nano chips (Agilent, Waldbronn, Germany). RNA quantity was determined photospectrometrically using a BioPhotometer 6131 (Eppendorf).

A total of 500 ng of DNA-free total RNA was converted into single-stranded using a mix of random and oligo-dT20 primers according to the ABgene protocol (ABgene, Hamburg, Germany). Real-time PCR oligonucleotide primers were designed using the online Primer3 internet based interface[72]. Primers were designed by the rules of highest maximum efficiency and sensitivity rules were followed to avoid formation of self and hetero-dimers, hairpins and self-complementarity (see Additional file 2). Gene-specific primers were designed on the basis of sequence obtained for selected *T. ni *genes and several additional genes as potential housekeeping genes to serve as the endogenous control (normalizer). Q-RT-PCR was performed in optical 96-well plates on a MX3000P Real-Time PCR Detection System (Stratagene, Amsterdam, The Netherlands) using the Absolute QPCR SYBR green Mix (ABgene) to monitor double-stranded DNA synthesis in combination with ROX as a passive reference dye included in the PCR master mix.

A dissociation curve analysis was performed for all primer/probe pairs, and all experimental samples yielded a single sharp peak at the amplicon's melting temperature. The dynamic range of a given primer/probe system and its normalizer was examined by running triplicate reactions of tenfold dilution series (five different RNA concentrations). As target and normalizer had similar dynamic ranges, the comparative quantitation method (ΔΔCt) was used to contrast the different treatments and tissues, and transformed to absolute values with 2^-ΔΔCt ^for obtaining relative fold changes [73]. All of the assays were run in quadruplicate (biological replication, each representing a pooled mRNA of four individuals) and triplicate (technical replication) to control for overall variability. Relative fold changes for each gene were set to 1 for the control treatment (larvae grown on bacteria-free diet).

### Statistical analysis

Statistical analyses were performed with the software package Statistica 7 (StatSoft, Hamburg, Germany). Normality of the data was estimated by using Shapiro-Wilkoxon and Levene's tests. In the case where assumptions for normality and homogeneity were not violated, our hypotheses were tested using an ANOVA model, otherwise a nonparametric Kruskall-Wallis ANOVA test was used.

## Authors' contributions

DF carried out the molecular laboratory work, with the help of HV, and performed the statistical analysis of the data. HV created the cDNA libraries and participated in the analysis of RT-qPCR data. DF and HV did the conception and design of the study and CW and DH participated in its design and helped to draft the manuscript. All authors participated in the writing and approval of the final manuscript.

## Supplementary Material

Additional file 1 Peptides from 1D SDS-PAGE gel-isolated proteins identified by MS. Click here for file

Additional file 2 Table with RT-qPCR primers used in this paper. Click here for file
