# Supplementary material for: Immune system responses and fitness costs associated with consumption of bacteria in larvae of Trichoplusia ni
Source: BMC Biol. 2007 Dec 21;5:56. doi: 10.1186/1741-7007-5-56 (PMC2235825; doi:10.1186/1741-7007-5-56)
Supplement: Additional file 2 — Table with RT-qPCR primers used in this paper. [file 1741-7007-5-56-S2.DOC]

**Additional File 2.** Real-time quantitative PCR primers used in this study

| **Gene** | **Forward Primer Sequence 5`-3`** | **Reverse Primer Sequence 5`-3`** |
| --- | --- | --- |
| Elongation init. factor 4 alpha (control) | GTGAGCGCGAAGTTATTATGC | AGAAACTTGCTGCACGTCAAT |
| Apolipophorin III | TCGTTAAGGAAGTAGCCAGCA | AGTTTCTTCTGCACCTGTTC |
| Arylphorin | GAACAACTCGACCTTCACTCG | AGCCTCATTCCTCATCCTTTC |
| Kalicitin | GATGATCGACGCTAGAGATGC | CAGAATTTCCGGTCTCACTGT |
| Azurocidin | GAAGGCGGTCCTTTAGTATGC | GGTAGCCGATGATGACGTAGA |
| Lebocin | GAGGGTTGAGAGGAGTCTTGG | TTAGGAACGAATGGAGGTGTG |
| BGRP | AACCAGACGGCATCGAATTAT | GAAGACCAAGGGTCACATGAA |
| Hemolin | TTCTGTTCAGGGCTGACAACT | GAGCAACCTCTTTCCTCCAGT |
| Cecropin A | TTCGCTTGTCTGGTCTTCACT | CACGAATGTTCTGTCCAACCT |
| Cecropin B | ATATGAATTTCTCCCGCGTGT | GCCTTGATGATACCGTCTCTG |
| Cecropin D | ATTTGGAAGGAATTGGTCAGC | CTTATTCCCTTTGCTGCTGCT |
| Cobatoxin | TTACATCCCAATCCTGACGAG | CGGATGATGGTAGATAAGGTAGG |
| Defensin | CAATAAGCAGTGAAGCCTTGG | GCATATGCCGTAGTTGTAGCC |
| Gallerimycin | TGCATTGCCAGTTGTAGACAG | ATAGCCTCAAGCTCATCACCA |
| Gloverin | CTTGATGTCCACAAGCAGGTT | CAAAGGTCTTGTCCAGATTGC |
| HDD1 | GGCTATACTACCGCGAGATCC | CGCCATTGTGGTCTTCTCTTA |
| Lysozyme (a) | ATGCGCCAAGAAGATCTACAA | GTTTAGCATTTGCTGATGTCG |
| Lysozyme (b) | TGTAGCAAGACCAGCACTCCT | CCTGGAACTTGTGACGTTTGT |
| PO inhib. enzyme | TGGCCTTCGTCTTCAAACTTA | CGACAGAACAACACGTTTACC |
| ProPO activating enzyme | AAGTCGGAAGAAGAGGTCGAG | CTGGCGTGTAACATGATCCTT |
| Ribosomal protein S18 (control) | TGTCCTATTTGTCGGGATGAG | TGGTCCCATGCTCTTTCTATG |
| Attacin | GGGAACCAAACTTCGGACTTA | CAGAGTCGGTGGCTACCTATAAA |
| GNBP | CGCTATGGTCGTGTTGAAGTT | CTGGATGTCAAGGTCATCGTT |
| BGBP | GCACTGGGCTAAAGGAACTGT | ATCGGGAAATTCGTTGATACC |
| Apolipophorin 3 (b) | GCACAATGGCAGCTAAGTTGT | GGTTGGTTCTTCGGAGTAAGG |
| Arylphorin (b) | AACTAGCTTCGTGATCCGTCA | TTAACTGGGTATCCGTTGCTG |
| ProPO activating enzyme (b) | ATGCATCGCGAAATACAGAAC | ATGCCTTCAGGCTTTCTCTTC |
| Elongation factor 1 alpha (control) | CGGTCAAATCTCAAACGGATA | CGTCACCAGACTTGATGGATT |
